# 1-(4-Chloro­butano­yl)-3-(2-chloro­phen­yl)thio­urea

**DOI:** 10.1107/S160053681201759X

**Published:** 2012-04-25

**Authors:** M. Sukeri M. Yusof, Nur Farhana Embong, Bohari M. Yamin, Nurziana Ngah

**Affiliations:** a Department of Chemical Sciences, Faculty of Science and Technology, Universiti Malaysia Terengganu, Menggabang Telipot, 21030 Kuala Terengganu, Malaysia; b School of Chemical Sciences and Food Technology, Universiti Kebangsaan Malaysia, UKM 43600 Bangi Selangor, Malaysia; c Kulliyyah of Science, International Islamic University Malaysia, Bandar Indera Mahkota, 25200 Kuantan, Pahang, Malaysia

## Abstract

The asymmetric unit of the title compound, C_11_H_12_Cl_2_N_2_OS, contains two crystallographically independent mol­ecules with different conformations: the benzene ring and the thio­urea fragment form dihedral angles of 74.32 (11) and 89.39 (11)°. One amino group in each mol­ecule is involved in intra­molecular N—H⋯O and inter­molecular N—H⋯O hydrogen bonding: the latter links pairs of independent mol­ecules into dimers. In the crystal, weak N—H⋯S inter­actions link these dimers into chains propagating along the *c* axis.

## Related literature

For a related structure, see: Yusof *et al.* (2011 ▶). For bond-length data, see: Allen *et al.* (1987 ▶).
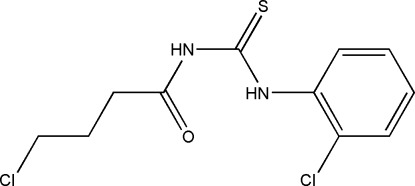


## Experimental

### 

#### Crystal data

C_11_H_12_Cl_2_N_2_OS
*M* *_r_* = 291.19
Monoclinic, 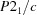

*a* = 14.396 (3) Å
*b* = 10.941 (2) Å
*c* = 18.093 (4) Å
β = 109.399 (4)°
*V* = 2688.0 (9) Å^3^
*Z* = 8
Mo *K*α radiation
μ = 0.62 mm^−1^
*T* = 298 K
0.42 × 0.41 × 0.39 mm

#### Data collection

Bruker SMART APEX CCD area-detector diffractometer
Absorption correction: multi-scan (*SADABS*; Bruker, 2000 ▶) *T*_min_ = 0.780, *T*_max_ = 0.793
14457 measured reflections
4727 independent reflections
3802 reflections with *I* > 2/s(*I*)
*R*_int_ = 0.023

#### Refinement

*R*[*F* ^2^ > 2σ(*F* ^2^)] = 0.039
*wR*(*F* ^2^) = 0.109
*S* = 1.03
4727 reflections
307 parameters
H-atom parameters constrained
Δρ_max_ = 0.40 e Å^−3^
Δρ_min_ = −0.33 e Å^−3^

### 

Data collection: *SMART* (Bruker, 2000 ▶); cell refinement: *SAINT* (Bruker, 2000 ▶); data reduction: *SAINT*; program(s) used to solve structure: *SHELXS97* (Sheldrick, 2008 ▶); program(s) used to refine structure: *SHELXL97* (Sheldrick, 2008 ▶); molecular graphics: *SHELXTL* (Sheldrick, 2008 ▶); software used to prepare material for publication: *SHELXTL*, *PARST* (Nardelli, 1995 ▶) and *PLATON* (Spek, 2009 ▶).

## Supplementary Material

Crystal structure: contains datablock(s) global, I. DOI: 10.1107/S160053681201759X/cv5287sup1.cif

Structure factors: contains datablock(s) I. DOI: 10.1107/S160053681201759X/cv5287Isup2.hkl

Supplementary material file. DOI: 10.1107/S160053681201759X/cv5287Isup3.cml

Additional supplementary materials:  crystallographic information; 3D view; checkCIF report

## Figures and Tables

**Table 1:** Hydrogen-bond geometry (Å, °)

*D*—H⋯*A*	*D*—H	H⋯*A*	*D*⋯*A*	*D*—H⋯*A*
N1—H1*A*⋯O1	0.86	2.01	2.675 (3)	133
N3—H3*A*⋯O2	0.86	1.98	2.652 (3)	134
N1—H1*A*⋯O2	0.86	2.37	3.070 (3)	138
N3—H3*A*⋯O1	0.86	2.37	3.073 (3)	139
N2—H2*A*⋯S2^i^	0.86	2.58	3.404 (2)	160
N4—H4*A*⋯S1^ii^	0.86	2.58	3.433 (2)	174

Symmetry codes: (i) 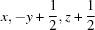; (ii) 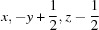.

**Fig. 1.**
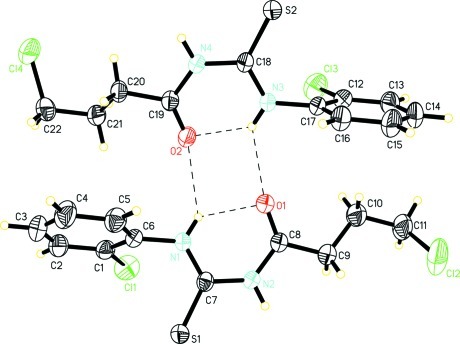
The content of asymmetric unit of the title compound showing the atomic numbering and 40% probability displacement ellipsoids. Dashed lines denote hydrogen bonds.

**Fig. 2.**
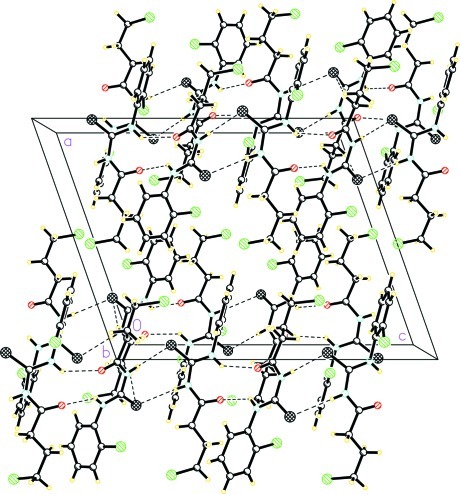
The molecular packing viewed down the b axis. Dashed lines denote N—H···S interactions.

## supplementary crystallographic information

### Comment

The asymmetric unit of the title compound, (I), contains two crystallographically independent molecules with different conformations. The title molecule is similiar to the previously reported *N*-(4-chlorobutanoyl)- *N*'-(2-fluorophenyl)thiourea (Yusof *et al.*, 2011) except the fluorine atom at *ortho* position of benzene ring is substituted by the chlorine atom at the same position. The bond lengths and angles are in normal ranges (Allen *et al.*, 1987). The benzene [(C1—C6) & (C12—C17)] and thiourea [(N1/N2/C7/S1/C6) & (N3/N4/C18/S2/C17)] fragments are each planar with maximum deviation is 0.047 (2) Å for atom N4 from the mean plane. In each independent molecule, the benzene and thiourea fragments make dihedral angles of 74.32 (11)° and 89.39 (11)°, respectively and comparable to those reported by Yusof *et al.*, (2011). One amino group in each molecule, N1—H1A and N3—H3A, respectively, is involved in bifurcated N—H···O hydrogen-bonding (Table 1) - intra and intermolecular ones, respectively, and the latter ones link two independent molecules into dimer. In the crystal, weak N—H···S interactions (Table 1) link further these dimers into chains propagated along *c* axis.

### Experimental

30 ml Acetone solution of 2-chloroanaline (1.81 g, 14.29 mmol) were added into a round-bottom flask containing a solution of 4-chlorobutanoylchloride (2.00 g, 14.29 mmol) and ammonium thiocyanate (1.10 g, 14.29 mmol). The solution mixture was refluxed for 2.5 h then filtered off and left to evaporate at room temperature. The yellowish precipitate obtained was washed with water and cold ethanol. The yellowish crystals suitable for X-ray analysis were obtained by recrystallization of the precipitate in DMF.

### Refinement

All H atoms were positioned geometrically [C—H 0.93–0.97 Å; N—H 0.86 Å], and refined using a riding model, with *U*_iso_(H) = 1.2 *U*_eq_(C, N).

### Crystal data

**Table:**

C_11_H_12_Cl_2_N_2_OS	*F*(000) = 1200
*M**_r_* = 291.19	*D*_x_ = 1.439 Mg m^−^^3^
Monoclinic, *P*2_1_/*c*	Mo *K*α radiation, λ = 0.71073 Å
Hall symbol: -P 2ybc	Cell parameters from 6026 reflections
*a* = 14.396 (3) Å	θ = 1.5–25.0°
*b* = 10.941 (2) Å	µ = 0.62 mm^−^^1^
*c* = 18.093 (4) Å	*T* = 298 K
β = 109.399 (4)°	Block, yellow
*V* = 2688.0 (9) Å^3^	0.42 × 0.41 × 0.39 mm
*Z* = 8	

### Data collection

**Table:**

Bruker SMART APEX CCD area-detector diffractometer	4727 independent reflections
Radiation source: fine-focus sealed tube	3802 reflections with *I* > 2σ(*I*)
Graphite monochromator	*R*_int_ = 0.023
Detector resolution: 83.66 pixels mm^-1^	θ_max_ = 25.0°, θ_min_ = 1.5°
ω scan	*h* = −17→17
Absorption correction: multi-scan (*SADABS*; Bruker, 2000)	*k* = −13→12
*T*_min_ = 0.780, *T*_max_ = 0.793	*l* = −16→21
14457 measured reflections	

### Refinement

**Table:**

Refinement on *F*^2^	Primary atom site location: structure-invariant direct methods
Least-squares matrix: full	Secondary atom site location: difference Fourier map
*R*[*F*^2^ > 2σ(*F*^2^)] = 0.039	Hydrogen site location: inferred from neighbouring sites
*wR*(*F*^2^) = 0.109	H-atom parameters constrained
*S* = 1.03	*w* = 1/[σ^2^(*F*_o_^2^) + (0.0514*P*)^2^ + 1.5167*P*] where *P* = (*F*_o_^2^ + 2*F*_c_^2^)/3
4727 reflections	(Δ/σ)_max_ < 0.001
307 parameters	Δρ_max_ = 0.40 e Å^−^^3^
0 restraints	Δρ_min_ = −0.33 e Å^−^^3^

### Special details

**Table:**

Geometry. All e.s.d.'s (except the e.s.d. in the dihedral angle between two l.s. planes) are estimated using the full covariance matrix. The cell e.s.d.'s are taken into account individually in the estimation of e.s.d.'s in distances, angles and torsion angles; correlations between e.s.d.'s in cell parameters are only used when they are defined by crystal symmetry. An approximate (isotropic) treatment of cell e.s.d.'s is used for estimating e.s.d.'s involving l.s. planes.
Refinement. Refinement of *F*^2^ against ALL reflections. The weighted *R*-factor *wR* and goodness of fit *S* are based on *F*^2^, conventional *R*-factors *R* are based on *F*, with *F* set to zero for negative *F*^2^. The threshold expression of *F*^2^ > σ(*F*^2^) is used only for calculating *R*-factors(gt) *etc*. and is not relevant to the choice of reflections for refinement. *R*-factors based on *F*^2^ are statistically about twice as large as those based on *F*, and *R*- factors based on ALL data will be even larger.

### Fractional atomic coordinates and isotropic or equivalent isotropic displacement parameters (Å^2^)

**Table:**

	*x*	*y*	*z*	*U*_iso_*/*U*_eq_	
S1	−0.02869 (4)	−0.00674 (6)	0.33632 (3)	0.04643 (17)	
S2	0.23770 (4)	0.49698 (6)	0.02320 (4)	0.04616 (17)	
Cl1	−0.08277 (6)	−0.17066 (6)	0.15579 (5)	0.0684 (2)	
Cl2	0.52142 (7)	0.18550 (11)	0.47501 (6)	0.1086 (4)	
Cl3	0.38361 (6)	0.18324 (8)	0.09743 (5)	0.0738 (2)	
Cl4	−0.23405 (6)	0.18796 (9)	−0.20368 (5)	0.0792 (3)	
O1	0.20202 (12)	0.12434 (18)	0.23359 (10)	0.0555 (5)	
O2	0.05946 (12)	0.18451 (16)	0.07274 (10)	0.0543 (5)	
N1	0.01342 (13)	0.07137 (17)	0.21195 (10)	0.0385 (4)	
H1A	0.0562	0.0916	0.1904	0.046*	
N2	0.14546 (13)	0.04033 (17)	0.32535 (11)	0.0400 (4)	
H2A	0.1648	0.0114	0.3722	0.048*	
N3	0.21846 (13)	0.32495 (18)	0.11973 (11)	0.0430 (5)	
H3A	0.1852	0.2670	0.1311	0.052*	
N4	0.08539 (13)	0.35124 (17)	0.00731 (11)	0.0382 (4)	
H4A	0.0585	0.3957	−0.0334	0.046*	
C1	−0.14018 (18)	−0.0300 (2)	0.13660 (14)	0.0450 (6)	
C2	−0.2387 (2)	−0.0240 (3)	0.09106 (16)	0.0620 (8)	
H2	−0.2740	−0.0952	0.0724	0.074*	
C3	−0.2839 (2)	0.0878 (3)	0.07364 (18)	0.0734 (9)	
H3	−0.3500	0.0921	0.0431	0.088*	
C4	−0.2326 (2)	0.1928 (3)	0.10078 (19)	0.0683 (8)	
H4	−0.2636	0.2682	0.0878	0.082*	
C5	−0.13441 (19)	0.1870 (2)	0.14771 (16)	0.0540 (6)	
H5	−0.0997	0.2584	0.1669	0.065*	
C6	−0.08857 (16)	0.0750 (2)	0.16572 (13)	0.0394 (5)	
C7	0.04504 (15)	0.03795 (19)	0.28697 (13)	0.0358 (5)	
C8	0.21855 (16)	0.0824 (2)	0.29900 (14)	0.0437 (5)	
C9	0.31985 (18)	0.0696 (3)	0.35824 (16)	0.0608 (7)	
H9A	0.3219	0.1128	0.4056	0.073*	
H9B	0.3322	−0.0161	0.3715	0.073*	
C10	0.39933 (19)	0.1170 (3)	0.33112 (17)	0.0644 (8)	
H10A	0.3885	0.2036	0.3200	0.077*	
H10B	0.3955	0.0764	0.2825	0.077*	
C11	0.5010 (2)	0.0994 (4)	0.38907 (18)	0.0743 (9)	
H11A	0.5491	0.1226	0.3647	0.089*	
H11B	0.5107	0.0135	0.4027	0.089*	
C12	0.39626 (18)	0.2933 (2)	0.16893 (14)	0.0487 (6)	
C13	0.48814 (19)	0.3193 (3)	0.22162 (17)	0.0610 (7)	
H13	0.5437	0.2795	0.2183	0.073*	
C14	0.4967 (2)	0.4046 (3)	0.27902 (18)	0.0690 (8)	
H14	0.5584	0.4223	0.3149	0.083*	
C15	0.4150 (2)	0.4644 (3)	0.28422 (18)	0.0700 (8)	
H15	0.4217	0.5219	0.3235	0.084*	
C16	0.32314 (19)	0.4390 (3)	0.23107 (16)	0.0562 (7)	
H16	0.2679	0.4798	0.2343	0.067*	
C17	0.31345 (16)	0.3533 (2)	0.17331 (13)	0.0435 (6)	
C18	0.17954 (15)	0.3847 (2)	0.05298 (12)	0.0355 (5)	
C19	0.02925 (16)	0.2559 (2)	0.01862 (13)	0.0398 (5)	
C20	−0.07275 (16)	0.2507 (2)	−0.03992 (15)	0.0455 (6)	
H20A	−0.0723	0.2884	−0.0883	0.055*	
H20B	−0.1165	0.2979	−0.0202	0.055*	
C21	−0.11259 (18)	0.1228 (2)	−0.05729 (15)	0.0527 (6)	
H21A	−0.1075	0.0822	−0.0084	0.063*	
H21B	−0.0726	0.0776	−0.0817	0.063*	
C22	−0.2184 (2)	0.1201 (3)	−0.11039 (17)	0.0689 (8)	
H22A	−0.2588	0.1636	−0.0854	0.083*	
H22B	−0.2410	0.0360	−0.1180	0.083*	

### Atomic displacement parameters (Å^2^)

**Table:**

	*U*^11^	*U*^22^	*U*^33^	*U*^12^	*U*^13^	*U*^23^
S1	0.0392 (3)	0.0612 (4)	0.0382 (3)	−0.0085 (3)	0.0118 (3)	−0.0020 (3)
S2	0.0413 (3)	0.0525 (4)	0.0396 (3)	−0.0108 (3)	0.0067 (3)	0.0051 (3)
Cl1	0.0886 (5)	0.0397 (4)	0.0721 (5)	−0.0045 (3)	0.0203 (4)	−0.0071 (3)
Cl2	0.0732 (6)	0.1360 (9)	0.0916 (7)	−0.0178 (6)	−0.0060 (5)	−0.0378 (6)
Cl3	0.0698 (5)	0.0801 (5)	0.0665 (5)	0.0138 (4)	0.0159 (4)	−0.0065 (4)
Cl4	0.0651 (5)	0.0973 (6)	0.0554 (4)	−0.0125 (4)	−0.0066 (4)	0.0048 (4)
O1	0.0387 (9)	0.0765 (12)	0.0466 (10)	−0.0065 (9)	0.0076 (8)	0.0167 (9)
O2	0.0442 (9)	0.0552 (10)	0.0505 (10)	−0.0112 (8)	−0.0018 (8)	0.0148 (9)
N1	0.0307 (9)	0.0449 (11)	0.0362 (10)	−0.0047 (8)	0.0062 (8)	0.0032 (8)
N2	0.0336 (10)	0.0486 (11)	0.0332 (10)	−0.0001 (8)	0.0050 (8)	0.0053 (8)
N3	0.0321 (10)	0.0524 (12)	0.0382 (10)	−0.0086 (8)	0.0031 (8)	0.0081 (9)
N4	0.0333 (9)	0.0435 (10)	0.0326 (9)	−0.0016 (8)	0.0039 (8)	0.0036 (8)
C1	0.0467 (13)	0.0463 (13)	0.0381 (13)	−0.0081 (11)	0.0089 (11)	−0.0007 (10)
C2	0.0508 (15)	0.077 (2)	0.0480 (15)	−0.0241 (15)	0.0033 (13)	−0.0025 (14)
C3	0.0405 (15)	0.104 (3)	0.0628 (19)	−0.0010 (17)	−0.0006 (13)	0.0162 (18)
C4	0.0504 (16)	0.073 (2)	0.072 (2)	0.0178 (15)	0.0067 (15)	0.0119 (16)
C5	0.0520 (15)	0.0462 (14)	0.0574 (16)	0.0027 (12)	0.0095 (13)	0.0021 (12)
C6	0.0350 (11)	0.0460 (13)	0.0337 (11)	−0.0014 (10)	0.0066 (9)	0.0044 (10)
C7	0.0345 (11)	0.0316 (11)	0.0374 (12)	−0.0011 (9)	0.0067 (9)	−0.0026 (9)
C8	0.0360 (12)	0.0510 (14)	0.0399 (13)	−0.0020 (10)	0.0071 (10)	0.0040 (11)
C9	0.0411 (14)	0.087 (2)	0.0479 (15)	−0.0073 (14)	0.0059 (12)	0.0124 (14)
C10	0.0419 (14)	0.093 (2)	0.0562 (17)	0.0013 (14)	0.0129 (13)	0.0058 (16)
C11	0.0378 (14)	0.113 (3)	0.0657 (19)	0.0026 (16)	0.0092 (13)	−0.0008 (18)
C12	0.0416 (13)	0.0552 (15)	0.0441 (14)	−0.0034 (11)	0.0074 (11)	0.0109 (11)
C13	0.0343 (13)	0.0751 (19)	0.0650 (18)	0.0009 (13)	0.0049 (12)	0.0169 (16)
C14	0.0412 (15)	0.084 (2)	0.0635 (19)	−0.0182 (15)	−0.0068 (13)	0.0083 (17)
C15	0.0652 (19)	0.075 (2)	0.0554 (18)	−0.0198 (16)	0.0008 (15)	−0.0108 (15)
C16	0.0448 (14)	0.0633 (17)	0.0530 (16)	−0.0035 (12)	0.0065 (12)	−0.0016 (13)
C17	0.0339 (12)	0.0532 (14)	0.0364 (12)	−0.0063 (10)	0.0024 (10)	0.0096 (11)
C18	0.0302 (10)	0.0407 (12)	0.0327 (11)	0.0019 (9)	0.0066 (9)	−0.0030 (9)
C19	0.0346 (11)	0.0408 (12)	0.0403 (13)	−0.0014 (10)	0.0073 (10)	−0.0017 (10)
C20	0.0321 (11)	0.0481 (14)	0.0492 (14)	−0.0023 (10)	0.0041 (10)	0.0025 (11)
C21	0.0456 (14)	0.0565 (15)	0.0486 (15)	−0.0126 (12)	0.0058 (12)	0.0036 (12)
C22	0.0501 (16)	0.089 (2)	0.0587 (17)	−0.0276 (15)	0.0064 (13)	−0.0009 (16)

### Geometric parameters (Å, º)

**Table:**

S1—C7	1.671 (2)	C5—H5	0.9300
S2—C18	1.673 (2)	C8—C9	1.502 (3)
Cl1—C1	1.727 (3)	C9—C10	1.481 (4)
Cl2—C11	1.758 (3)	C9—H9A	0.9700
Cl3—C12	1.732 (3)	C9—H9B	0.9700
Cl4—C22	1.788 (3)	C10—C11	1.504 (4)
O1—C8	1.217 (3)	C10—H10A	0.9700
O2—C19	1.215 (3)	C10—H10B	0.9700
N1—C7	1.332 (3)	C11—H11A	0.9700
N1—C6	1.429 (3)	C11—H11B	0.9700
N1—H1A	0.8600	C12—C13	1.379 (4)
N2—C8	1.371 (3)	C12—C17	1.386 (3)
N2—C7	1.381 (3)	C13—C14	1.371 (4)
N2—H2A	0.8600	C13—H13	0.9300
N3—C18	1.323 (3)	C14—C15	1.375 (5)
N3—C17	1.423 (3)	C14—H14	0.9300
N3—H3A	0.8600	C15—C16	1.381 (4)
N4—C19	1.376 (3)	C15—H15	0.9300
N4—C18	1.382 (3)	C16—C17	1.377 (4)
N4—H4A	0.8600	C16—H16	0.9300
C1—C6	1.374 (3)	C19—C20	1.499 (3)
C1—C2	1.385 (4)	C20—C21	1.505 (4)
C2—C3	1.371 (5)	C20—H20A	0.9700
C2—H2	0.9300	C20—H20B	0.9700
C3—C4	1.365 (5)	C21—C22	1.507 (4)
C3—H3	0.9300	C21—H21A	0.9700
C4—C5	1.388 (4)	C21—H21B	0.9700
C4—H4	0.9300	C22—H22A	0.9700
C5—C6	1.379 (3)	C22—H22B	0.9700
			
C7—N1—C6	122.78 (18)	C10—C11—Cl2	112.7 (2)
C7—N1—H1A	118.6	C10—C11—H11A	109.1
C6—N1—H1A	118.6	Cl2—C11—H11A	109.1
C8—N2—C7	128.89 (19)	C10—C11—H11B	109.1
C8—N2—H2A	115.6	Cl2—C11—H11B	109.1
C7—N2—H2A	115.6	H11A—C11—H11B	107.8
C18—N3—C17	122.27 (19)	C13—C12—C17	120.6 (3)
C18—N3—H3A	118.9	C13—C12—Cl3	119.9 (2)
C17—N3—H3A	118.9	C17—C12—Cl3	119.53 (19)
C19—N4—C18	128.30 (19)	C14—C13—C12	119.2 (3)
C19—N4—H4A	115.8	C14—C13—H13	120.4
C18—N4—H4A	115.8	C12—C13—H13	120.4
C6—C1—C2	120.3 (2)	C13—C14—C15	120.8 (3)
C6—C1—Cl1	120.41 (18)	C13—C14—H14	119.6
C2—C1—Cl1	119.3 (2)	C15—C14—H14	119.6
C3—C2—C1	119.5 (3)	C14—C15—C16	120.0 (3)
C3—C2—H2	120.3	C14—C15—H15	120.0
C1—C2—H2	120.3	C16—C15—H15	120.0
C4—C3—C2	120.6 (3)	C17—C16—C15	119.9 (3)
C4—C3—H3	119.7	C17—C16—H16	120.1
C2—C3—H3	119.7	C15—C16—H16	120.1
C3—C4—C5	120.1 (3)	C16—C17—C12	119.6 (2)
C3—C4—H4	120.0	C16—C17—N3	119.9 (2)
C5—C4—H4	120.0	C12—C17—N3	120.5 (2)
C6—C5—C4	119.7 (3)	N3—C18—N4	116.67 (19)
C6—C5—H5	120.2	N3—C18—S2	123.45 (16)
C4—C5—H5	120.2	N4—C18—S2	119.88 (16)
C1—C6—C5	119.8 (2)	O2—C19—N4	122.5 (2)
C1—C6—N1	121.4 (2)	O2—C19—C20	123.3 (2)
C5—C6—N1	118.8 (2)	N4—C19—C20	114.20 (19)
N1—C7—N2	116.99 (19)	C19—C20—C21	113.5 (2)
N1—C7—S1	124.22 (16)	C19—C20—H20A	108.9
N2—C7—S1	118.80 (16)	C21—C20—H20A	108.9
O1—C8—N2	122.7 (2)	C19—C20—H20B	108.9
O1—C8—C9	124.0 (2)	C21—C20—H20B	108.9
N2—C8—C9	113.3 (2)	H20A—C20—H20B	107.7
C10—C9—C8	113.9 (2)	C20—C21—C22	112.7 (2)
C10—C9—H9A	108.8	C20—C21—H21A	109.1
C8—C9—H9A	108.8	C22—C21—H21A	109.1
C10—C9—H9B	108.8	C20—C21—H21B	109.1
C8—C9—H9B	108.8	C22—C21—H21B	109.1
H9A—C9—H9B	107.7	H21A—C21—H21B	107.8
C9—C10—C11	113.9 (2)	C21—C22—Cl4	112.16 (19)
C9—C10—H10A	108.8	C21—C22—H22A	109.2
C11—C10—H10A	108.8	Cl4—C22—H22A	109.2
C9—C10—H10B	108.8	C21—C22—H22B	109.2
C11—C10—H10B	108.8	Cl4—C22—H22B	109.2
H10A—C10—H10B	107.7	H22A—C22—H22B	107.9
			
C6—C1—C2—C3	1.5 (4)	C17—C12—C13—C14	0.6 (4)
Cl1—C1—C2—C3	−178.0 (2)	Cl3—C12—C13—C14	−178.5 (2)
C1—C2—C3—C4	0.0 (5)	C12—C13—C14—C15	−0.3 (4)
C2—C3—C4—C5	−1.2 (5)	C13—C14—C15—C16	−0.2 (5)
C3—C4—C5—C6	1.0 (5)	C14—C15—C16—C17	0.5 (5)
C2—C1—C6—C5	−1.8 (4)	C15—C16—C17—C12	−0.2 (4)
Cl1—C1—C6—C5	177.8 (2)	C15—C16—C17—N3	178.4 (2)
C2—C1—C6—N1	−179.2 (2)	C13—C12—C17—C16	−0.4 (4)
Cl1—C1—C6—N1	0.4 (3)	Cl3—C12—C17—C16	178.7 (2)
C4—C5—C6—C1	0.5 (4)	C13—C12—C17—N3	−179.0 (2)
C4—C5—C6—N1	178.0 (2)	Cl3—C12—C17—N3	0.1 (3)
C7—N1—C6—C1	−76.9 (3)	C18—N3—C17—C16	89.7 (3)
C7—N1—C6—C5	105.7 (3)	C18—N3—C17—C12	−91.7 (3)
C6—N1—C7—N2	−178.95 (19)	C17—N3—C18—N4	−177.8 (2)
C6—N1—C7—S1	1.4 (3)	C17—N3—C18—S2	1.4 (3)
C8—N2—C7—N1	6.1 (3)	C19—N4—C18—N3	−6.3 (3)
C8—N2—C7—S1	−174.26 (19)	C19—N4—C18—S2	174.38 (18)
C7—N2—C8—O1	−0.9 (4)	C18—N4—C19—O2	−1.8 (4)
C7—N2—C8—C9	180.0 (2)	C18—N4—C19—C20	176.4 (2)
O1—C8—C9—C10	2.1 (4)	O2—C19—C20—C21	−33.5 (3)
N2—C8—C9—C10	−178.8 (3)	N4—C19—C20—C21	148.3 (2)
C8—C9—C10—C11	−177.6 (3)	C19—C20—C21—C22	174.7 (2)
C9—C10—C11—Cl2	−66.2 (4)	C20—C21—C22—Cl4	61.0 (3)

### Hydrogen-bond geometry (Å, º)

**Table:**

*D*—H···*A*	*D*—H	H···*A*	*D*···*A*	*D*—H···*A*
N1—H1*A*···O1	0.86	2.01	2.675 (3)	133
N3—H3*A*···O2	0.86	1.98	2.652 (3)	134
N1—H1*A*···O2	0.86	2.37	3.070 (3)	138
N3—H3*A*···O1	0.86	2.37	3.073 (3)	139
N2—H2*A*···S2^i^	0.86	2.58	3.404 (2)	160
N4—H4*A*···S1^ii^	0.86	2.58	3.433 (2)	174

Symmetry codes: (i) *x*, −*y*+1/2, *z*+1/2; (ii) *x*, −*y*+1/2, *z*−1/2.
